# Characterization of the spoilage microbial communities in plant-based meat alternatives during refrigerated storage

**DOI:** 10.1128/spectrum.01650-25

**Published:** 2025-09-12

**Authors:** Mehmet Dogan, David A. Mann, Xiangyu Deng

**Affiliations:** 1 Center for Food Safety, Department of Food Science and Technology, University of Georgia 92569, Griffin, Georgia, USA; 2 National Food Reference Laboratory, Yenimahalle, Türkiye; University of Mississippi, University, Mississippi, USA

**Keywords:** 16S rRNA, ITS, microbiota, spoilage, meat alternatives

## Abstract

Plant-based meat alternatives (PBMAs), primarily derived from pea or soy proteins, are gaining popularity and market share, yet their spoilage ecology remains understudied. To address this knowledge gap, we profiled the bacterial and fungal communities of 12 refrigerated PBMAs commonly sold in the United States (six pea-based meats [PBMs] and six soy-based meats [SBMs], each in ground and burger forms) during refrigerated shelf display and extended storage. Microbial loads rose rapidly and heterogeneously across products after in-store thawing and past sell-by dates, underscoring the need to handle PBMAs like any other highly perishable food, both in stores and at home. Soy and pea-based matrices exhibited distinct patterns of spoilage microbiota assemblage and succession, suggesting that the protein source shapes spoilage trajectories.

**IMPORTANCE:**

Our findings highlight that spoilage of plant-based meat alternatives (PBMAs) is shaped by the protein source, with soy- and pea-based products exhibiting distinct microbial trajectories and acidity profiles driven by both ingredient composition and spoilage microbiota. Identification of these ingredient-associated spoilage patterns lays the foundation for tailored and precision spoilage management for PBMAs.

## INTRODUCTION

Meat and meat products have been integral to well-balanced diets due to their nutritional richness, satiating qualities, and palatability. For ages, humans have sourced the majority of their protein from animals. In 2021, the average consumption of total meat, including beef, pork, and poultry, was 125 kg per capita per year in the USA and 67 kg per capita per year in the EU (1, 2). Global meat consumption in 2020 was around 34.1 kg per capita and is expected to reach 35.6 kg per capita by 2031 (3, 4).

Along with the increasing demand for animal protein, the meat industry faces environmental issues (such as greenhouse gas emissions, land use, and water use) (5, 6), ethical concerns (7), and health risks, including antibiotic resistance, foodborne illnesses, and diet-related diseases (8, 9). New, reliable, and readily accessible alternative protein sources are positioned to help meet the growing protein demand and address the various challenges faced by the meat industry.

Plant-based meat alternatives (PBMAs) have emerged as strong candidates for alternative sources of dietary protein (10, 11). PBMAs offer similar texture, appearance, and flavor to animal meat and are considered healthier than animal-derived counterparts, with benefits, such as a reduced risk of developing type II diabetes, cardiovascular diseases, and metabolic syndrome (12). They also have a lower environmental impact compared with conventional animal-based meat products (13). Moreover, PBMAs are becoming an increasingly significant part of consumers’ diets and are more accessible as the industry develops and manufactures a variety of alternatives (14, 15). As a result, the global market value of PBMAs is expected to grow from $6.42 billion in 2023 to $17.79 billion by 2032 (16).

Food spoilage is a significant challenge that impacts multiple components of the food chain, from manufacturing to consumption. It renders food products unsuitable for human consumption by altering sensory qualities, including appearance, texture, taste, and smell (17, 18). Microbial activity is estimated to account for nearly 30% of spoilage in manufactured food products, making it a primary contributor to food quality deterioration (19). Due to their neutral pH, high protein content, moisture, and relatively high-water activity, PBMAs provide an excellent environment for the proliferation of spoilage microorganisms and foodborne pathogens, similar to their meat counterparts (20–22). Although the high-temperature extrusion process kills most bacteria, some spore-forming bacteria, such as *Bacillus* spp. or *Clostridium* spp., may still endure the process (20, 21).

Moreover, recontamination may occur in PBMAs during post-thermal process handling, with *Latilactobacillus sakei*, *Enterococcus faecium*, and *Carnobacterium divergens* being among the most common bacterial contaminants (14, 20, 23). Mold and bacteria contaminations have been found in PBMA production environments (24). USDA researchers have emphasized that PBMA burgers should be handled like raw ground beef. They noted that PBMAs could carry foodborne pathogens if produced in a poor-quality manufacturing environment or with contaminated raw materials (25, 26). Additionally, it has been demonstrated that PBMAs support the growth of spoilage microorganisms, including *Pseudomonas fluorescens* and *Brochothrix thermosphacta*, as well as pathogenic microorganisms, including *Listeria monocytogenes*, *Escherichia coli* O157, and *Salmonella* spp. (25, 27).

Previous studies using 16S rRNA amplicon sequencing have characterized the microbiota of diverse PBMA products (28) and compared differences in spoilage-associated microbial communities between representative PBMA formulations and ground beef (29). This study aims to gain a deeper understanding of the temporal dynamics of the spoilage microbiota across a broad range of widely available PBMA products in the U.S. market, encompassing various protein sources (pea and soy) and product forms (ground and burger). Compared with previous studies, we simulated an extended spoilage process of PBMA products, encompassing both refrigerated in-store displays and subsequent refrigerated home storage. This process was designed to reflect a common scenario in which frozen products are thawed in retail stores, sold on refrigerated displays, and stored under refrigerated conditions by consumers beyond the sell-by date (30, 31).

## MATERIALS AND METHODS

### Sample acquisition

Twelve chilled PBMA samples, either pea-based meats (PBMs, Brand A) or soy-based meats (SBMs, Brand B), consisting of ground beef (lite or regular) and burgers, were purchased from local grocery stores and transferred to the University of Georgia’s Food Processing and Innovation Center. The PBMAs were stored in a walk-in cooler at 4°C either until their sell-by date (six samples) or up to 7 days beyond the sell-by date (six samples). According to the manufacturers' product labels, SBMs primarily consist of soy protein concentrate, coconut oil, and sunflower oil. SBM ground products were available in two forms: regular (Ground) and lite (Ground-Lite). PBMs are primarily made from pea protein, expeller-pressed canola oil, and refined coconut oil. PBM burgers had two forms that were a new size (Burger-N) and an old size (Burger). According to the store’s claims, the products were initially frozen and later thawed in-store on refrigerated shelves for sale. After thawing, store employees assigned them a 7-day sell-by date.

### Sample preparation

On sampling days, the sell-by date (Day 0) and 1 week after (Day 7), 50 g of PBMAs was aseptically weighed into Whirl-Pak Sterile Filter Bags (7.5″ × 12″) and combined with 50 mL of sterile buffered peptone water (BPW, Neogen). The samples were hand-massaged for 2 min to collect the rinsates. One and a half milliliters of rinsate from each PBMA sample was transferred into 1.6 mL Eppendorf tubes and stored at −20°C for later analysis.

### Microbiological analysis

The rinsates were also serially diluted (1:10) in BPW and plated on De Man, Rogosa, and Sharpe Agar (MRS, BD) (incubated at 30°C for 48 h) for lactic acid bacteria (LAB), as well as on Tryptic Soy Agar (TSA, BD) (incubated at 35°C for 48 h) for total aerobic plate counts. Microbial populations were enumerated either on the sell-by date or up to 7 days beyond it. Plate counts were performed in duplicate, whereas microbiota analysis was conducted with a single replicate.

### Physicochemical analysis

For each sample, a 1:10 dilution of a representative 5-g homogenized portion in 45-mL deionized water was prepared using a stomacher. The pH value of the homogenate was measured using a Mettler Toledo (FP20) benchtop pH meter with a pH ATC Combination Electrode (Mettler Toledo, pH Sensor LE 438). The water activity of PBMAs was measured using an AquaLab CX-2 water activity meter.

### DNA extraction and library preparation

A 1.5-mL aliquot of each PBMA rinsate was transferred into 2-mL collection tubes (Thermo Fisher, MA) for DNA extraction using the Qiagen PowerFood Microbial Kit (QIAGEN, Valencia, CA), following the manufacturer’s instructions. DNA purity and concentration were measured with a NanoDrop spectrophotometer (NanoDrop Technologies, DE, USA) at 260 and 280 nm. Additional DNA quantification was performed using the Qubit dsDNA High Sensitivity (HS) Assay Kit (Invitrogen, Thermo Fisher Scientific, Waltham, MA) with a Qubit 3.0 fluorometer (Life Technologies). From these, three samples of each protein type (totaling six samples per sampling point) were selected, resulting in 12 high-quality DNA samples for library preparation and sequencing.

The 16S rRNA and internal transcribed spacer (ITS) library preparation was performed according to Illumina’s 16S and ITS rRNA sequencing protocol, targeting the V3-V4 and ITS regions. Subsequently, the final libraries were sequenced on an Illumina MiSeq platform at the University of Georgia’s Center for Food Safety to obtain 300-bp paired-end reads.

### Sequence analysis

Sequences were demultiplexed on the Illumina MiSeq and were further processed using Qiime2 2024.5 version (32). Briefly, the raw FASTQ files were denoised using DADA2 (33) with default parameters. Taxonomy was assigned to the sequences by the QIIME2 q2-feature-classifier plugin (34). The pre-trained Naive Bayes classifier artifacts, based on the SILVA 16S rRNA gene database v138.2 (35, 36) obtained using RESCRIPt (37), along with the UNITE database (38), were utilized for the molecular identification of bacteria and fungi, respectively. The features with low abundances, such as singletons, and those with a total abundance of less than 10 (summed across all samples) were removed from the feature table. Sequences classified as Archaea, Eukaryota, chloroplasts, or mitochondria were removed. Align-to-tree-mafft-fasttree pipeline from the q2-phylogeny plugin was used to generate a phylogenetic tree (39, 40). Alpha-diversity metrics (observed OTUs and Shannon’s Diversity [41]), beta diversity metrics (Jaccard distance, and Bray-Curtis dissimilarity [42]), and principal coordinate analysis (PCoA) (43) were estimated using q2-diversity after samples were rarefied to 10,350 sequences per sample.

### Statistical analysis

Variations in microbial composition and structure across different sample types, forms, and storage durations were analyzed using the alpha-group-significance and beta-group-significance methods from the diversity plugin in QIIME 2. The alpha-group-significance method employed the Kruskal-Wallis test (44) to evaluate within-sample diversity metrics, such as Shannon and Observed Features. The Adonis PERMANOVA test, implemented in the vegan package in R, was used as a permutation-based statistical method to assess differences in beta diversity between groups using distance metrics such as Bray-Curtis, Jaccard, and Weighted Unifrac (45, 46). Community taxonomy composition, β-diversity, and α-diversity metrics were visualized using the R packages qiime2R (47), tidyverse (48), ggplot2 (49), and phyloseq (50). Statistical significance was determined at *P* < 0.05.

## RESULTS

During refrigerated storage, the bacterial spoilage microbiota in PBMA samples exhibited three distinct patterns: rapid succession in SBM-Ground and SBM-Burger, minor shifts in PBM-Ground, PBM-Burger-N, and SBM-Ground-Lite, and minimal change in PBM-Burger. Overall, PBMs exhibited higher pH values than SBMs, which in turn affected the composition and succession of dominant genera by the end of the storage period. Fungal communities were mostly stable, with only minor increases in low-abundance taxa. Microbiota clustered by protein type, with PBMs showing higher bacterial richness and SBMs showing higher fungal diversity.

### Temporal dynamics of bacterial communities during refrigerated storage in plant-based meat alternatives

**Fig 1:**
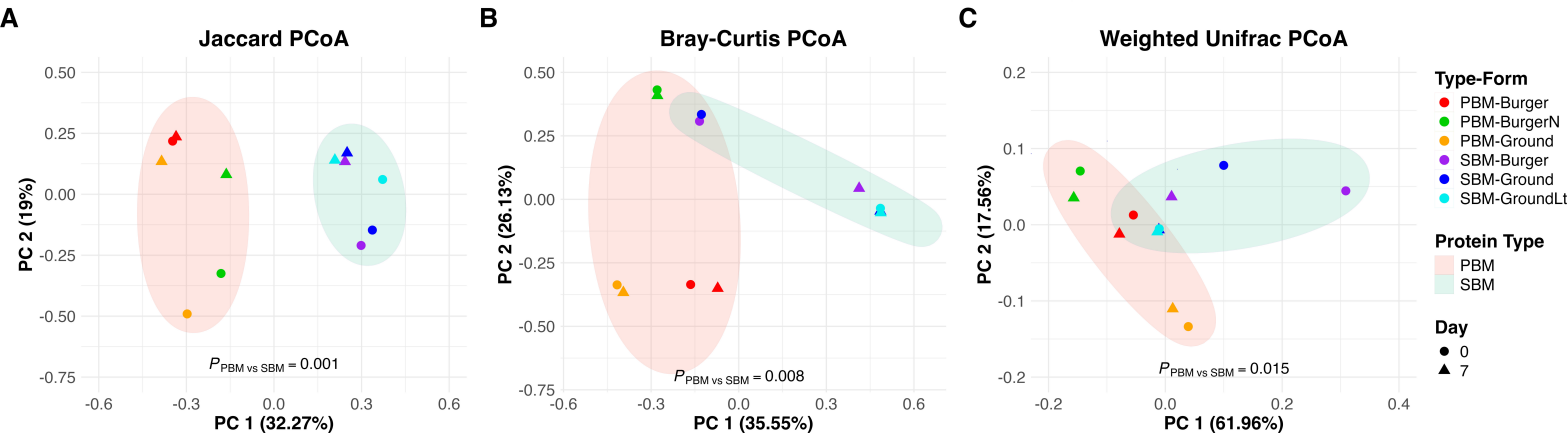
Principal coordinate analysis (PCoA) of bacterial communities in plant-based meat alternatives (PBMAs) stored and sampled on Day 0 (sell-by date) and Day 7 (7 days after the sell-by date) at 4°C. (**A**) Jaccard PCoA results for pea-based meats (PBMs) and soy-based meats (SBMs). (**B**) Bray-Curtis PCoA results for PBMs and SBMs. (**C**) Weighted Unifrac PCoA results for PBMs and SBMs. Pairwise comparisons were conducted using pairwise PERMANOVA.

Based on beta diversity indices, we identified three major patterns in the temporal shifts of spoilage bacteria. The first category, comprising SBM-Ground and SBM-Burger samples, exhibited pronounced changes across all three beta diversity indices (blue- and purple-colored samples in Fig. 1). These indices include Jaccard, which measures community dissimilarities based on taxa presence and absence alone while ignoring taxa abundance (Fig. 1A); Bray-Curtis, which accounts for differences in taxa abundances between microbial communities (Fig. 1B); and weighted UniFrac, which incorporates both taxa abundance and phylogenetic distances to compare microbial communities (Fig. 1C).

**Fig 2:**
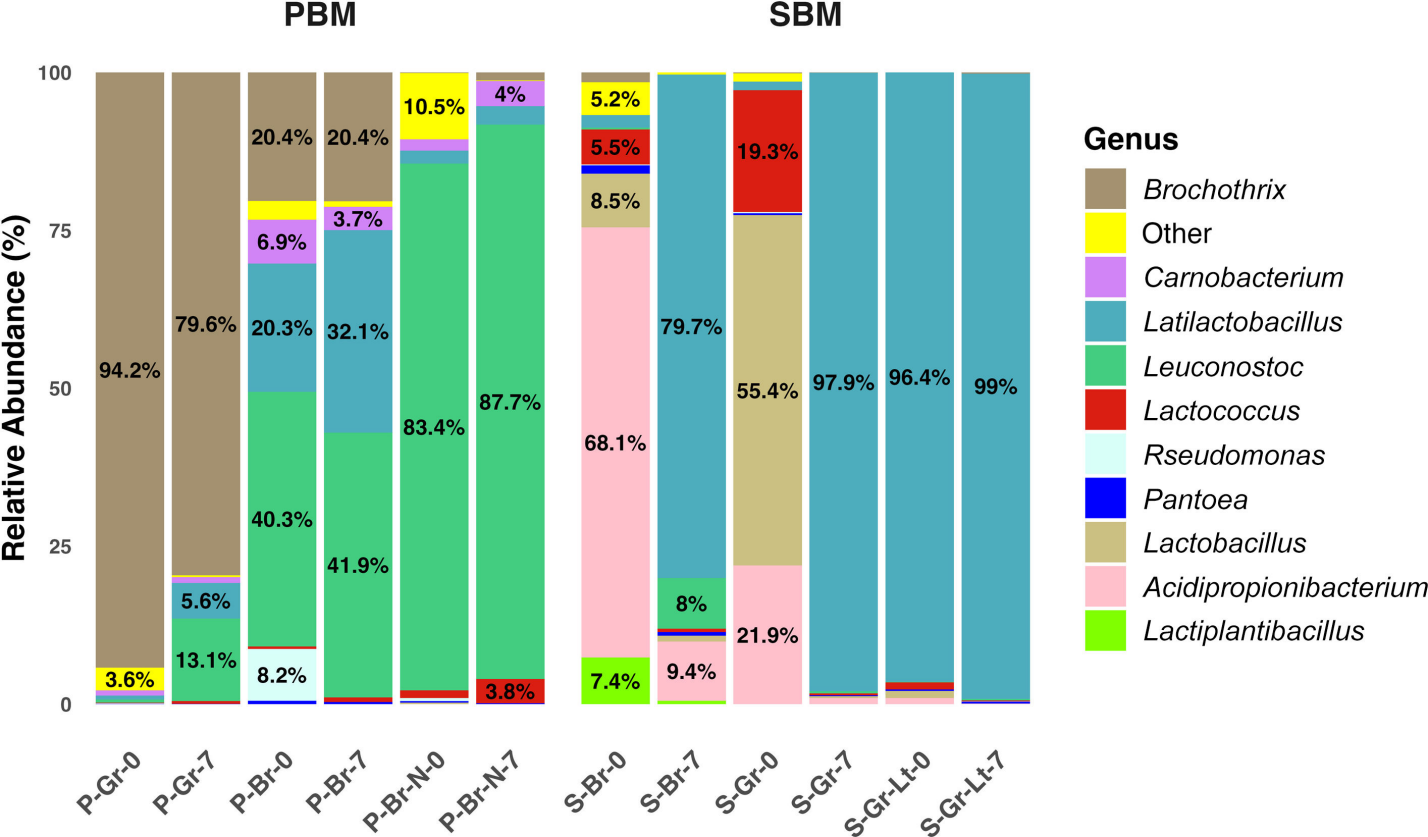
Indigenous microbial community composition of pea-based meat (PBM) and soy-based meat (SBM). Bacterial taxonomic classification at the genus level. Percentages are only shown for relative abundances of 3% or more. P: Pea. S: Soy. Gr: Ground. Br: Burger. N: New size. Lt: Lite. 0: Sell-by date. 7: 7 days after the sell-by date.

**Fig 3:**
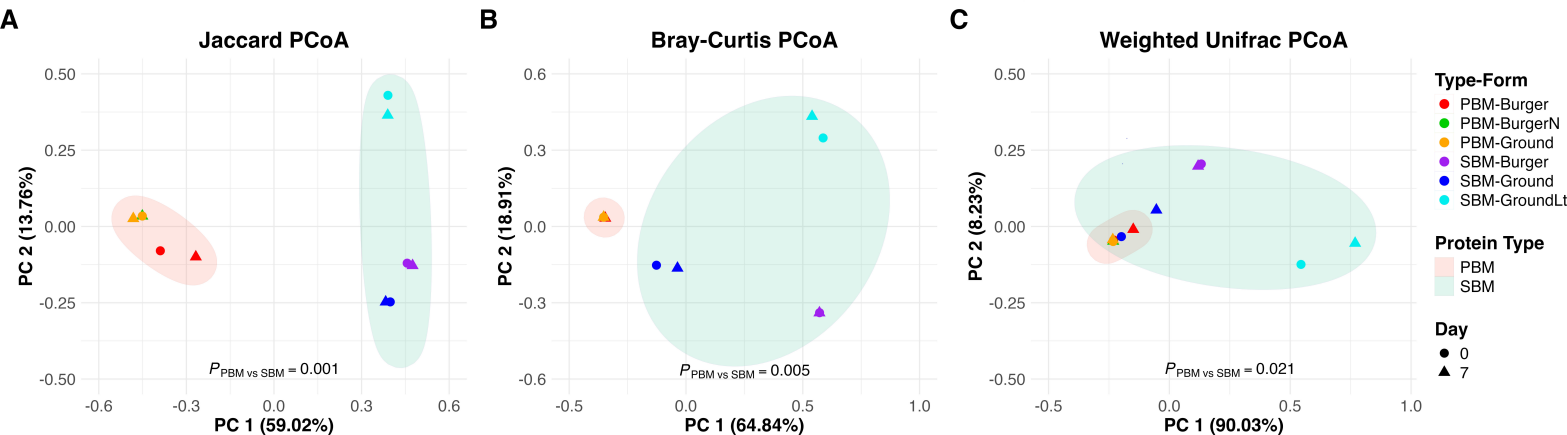
Principal coordinate analysis (PCoA) of fungal communities in plant-based meat alternatives (PBMAs) stored and sampled on Day 0 (sell-by date) and Day 7 (7 days after the sell-by date) at 4°C. (**A**) Jaccard PCoA results for pea-based meats (PBMs) and soy-based meats (SBMs). (**B**) Bray-Curtis PCoA results for PBMs and SBMs. (**C**) Weighted Unifrac PCoA results for PBMs and SBMs. Pairwise comparisons were conducted using pairwise PERMANOVA.

At the genus level, the bacterial compositions of SBM-Burger and SBM-Ground on the sell-by dates (Day 0) were similar, as indicated by their close placement in PCoA analyses based on both Jaccard (Fig. 1A) and Bray-Curtis (Fig. 1B) indices. However, the two samples differed notably by their most abundant genera on Day 0, with *Acidipropionibacterium* dominating in SBM-Burger (68.1%) and *Lactobacillus* dominating in SBM-ground (55.4%) (Fig. 2). Since these dominant genera belong to different phyla (*Actinomycetota* and *Bacillota*), their large phylogenetic distance separated the two samples in PCoA analysis using weighted UniFrac (Fig. 1C). Despite the initial difference, *Latilactobacillus* became the dominant genus in both bacterial communities as spoilage progressed, increasing from 2% to 79.7% in SBM-Burger and from 1.4% to 97.9% in SBM-Ground (Fig. 2). In contrast, the relative abundance of *Leuconostoc* increased from 1% to 8%, while *Acidiopropionibacterium* decreased from 68.1% to 9.4% in SBM-Burger samples. Additionally, the relative abundances of *Lactobacillus* (8.5%), *Lactiplantibacillus* (7.4%), *Lactococcus* (5.5%), *Brochothrix* (1.6%), *Pantoea* (1.3%), and “Other” taxa (5.2%) each declined to below 1% by Day 7. In SBM-Ground samples, *Lactobacillus* (55.4%), *Acidiopropionibacterium* (21.9%), *Lactococcus* (19.3%), and “Other” taxa (1.3%) were all present at <1% relative abundance on Day 7. Consistent with the observed temporal shifts in beta indices, both samples underwent substantial bacterial growth over 7 days. In SBM-Burger, LAB increased by 3.13 log CFU/g and APC by 2.98 log CFU/g. In SBM-Ground, LAB rose by 4.24 log CFU/g and APC by 5.29 log CFU/g. The initial bacterial loads in the two samples were approximately 4 log CFU/g, the lowest among all the tested samples (Fig. 3A).

The second category includes PBM-Ground, PBM-Burger-N, and SBM-Ground-Lite (orange, green, and cyan samples, respectively, in Fig. 1). The spoilage bacteria of these samples displayed pronounced temporal changes based on the Jaccard index (Fig. 1A) but showed minimal shifts according to both Bray-Curtis (Fig. 1B) and Weighted UniFrac indices (Fig. 1C). This observation suggests that the population dynamics of spoilage bacteria in these samples were driven by relatively less abundant taxa, while the dominant taxa remained stable (Fig. 2). The most abundant genera in these samples on Day 0 remained dominant on Day 7, with minor shifts in their relative abundances, including *Brochothrix* in PBM-Ground (from 94.2% to 79.6%), *Leuconostoc* in PBM-Burger-N increased from 83.4% to 87.7%, and *Latilactobacillus* in SBM-Ground-Lt rose from 96.4% to 99%. In contrast, the relative abundances of minor genera changed substantially. In PBM-Ground, *Leuconostoc* and *Latilactobacillus* increased from 0.5% and 0.6% on Day 0 to 13.1% and 5.6% on Day 7, respectively. In PBM-Burger-N, *Carnobacterium*, *Latilactobacillus*, and *Lactococcus* increased from 1.8%, 2.1%, and 1.2% to 4%, 2.9%, and 3.8%, respectively. In SBM-Ground-Lt, the total relative abundance of the minor genera, including *Lactococcus*, *Lactobacillus*, and *Acidipropionibacterium,* decreased from 3.4% to 1%. Meanwhile, *Lactobacillus* disappeared entirely, while *Brochothrix* and *Pseudomonas* emerged. Notably, the initial bacterial loads of these samples were at intermediate levels, ranging from 5.89 to 7.39 log CFU/g by LAB and 6.07 log CFU/g to 7.37 log CFU/g by APC, among all samples (Table 1).

**TABLE 1:** Changes in physicochemical properties and bacterial populations of commercial plant-based meat alternatives stored at 4°C: Comparison between the sell-by date and 7 days after^*a*^^,*d*^

Protein type/ Brand	Processing Facilities	Sample form - Sampling day	APC^*b*^	LAB^*c*^	pH	a_w_
PBM (Brand A)	A	Ground - 0	6.14 + 0.01	5.89 + 0.01	7.34 + 0.01	0.989 + 0.001
		Ground - 7	8.17 + 0.30	8.77 + 0.04	6.10 + 0.01	0.988 + 0.001
	B	Burger N - 0	6.07 + 0.01	6.08 + 0.06	7.15 + 0.01	0.995 + 0.001
		Burger N - 7	9.03 + 0.06	9.15 + 0.06	6.08 + 0.01	0.991 + 0.001
	A	Burger - 0	8.16 + 0.33	8.67 + 0.16	6.55 + 0.01	0.993 + 0.001
		Burger - 7	9.08 + 0.09	9.28 + 0.03	5.95 + 0.01	0.989 + 0.001
		**Mean - 0**	**6.79 + 1.19**	**6.88 + 1.55**	**7.01 + 0.41**	**0.992 + 0.003**
		**Mean - 7**	**8.76 + 0.51**	**9.07 + 0.27**	**6.04 + 0.08**	**0.989 + 0.002**
SBM (Brand B)	C	Burger - 0	4.07 + 0.06	4.08 + 0.05	6.12 + 0.01	0.990 + 0.001
		Burger - 7	7.05 + 0.09	7.20 + 0.03	6.08 + 0.01	0.987 + 0.001
	D	Ground - 0	3.38 + 0.09	4.56 + 0.02	6.13 + 0.01	0.986 + 0.001
		Ground - 7	8.67 + 0.01	8.80 + 0.01	5.66 + 0.02	0.987 + 0.001
	D	Ground Lite - 0	7.37 + 0.02	7.39 + 0.01	6.18 + 0.01	0.987 + 0.001
		Ground Lite - 7	8.86 + 0.01	9.00 + 0.06	5.21 + 0.01	0.990 + 0.001
		**Mean - 0**	**4.94 + 2.13**	**5.34 + 1.79**	**6.14 + 0.03**	**0.988 + 0.002**
		**Mean - 7**	**8.19 + 0.99**	**8.33 + 0.99**	**5.65 + 0.44**	**0.988 + 0.002**

^*a*^ PBM: Pea-based meat. SBM: Soy-based meat. Day 0: Sell-by date. Day 7: 7 days after the sell-by date. N: New size.

^*b*^ APC: aerobic plate counts.

^*c*^ LAB: lactic acid bacteria populations.

^*d*^ Bold indicates the mean: the average of values for each sampling day, including plate counts, pH, and a_w_.

The final category consists of a single sample, PBM-Burger, whose spoilage bacterial communities exhibited marginal temporal change across all three beta indices (red samples in Fig. 1). Except for *Pseudomonas*, which was present on Day 0 but undetected on Day 7, all major genera persisted through the 7 days. The relative abundances of the most abundant genera remained stable over the 7 days, with *Latilactobacillus* increasing from 20.3% to 32.1%, *Leuconostoc* increasing slightly from 40.3% to 41.9%, and *Brochothrix* remaining unchanged at 20.4%. As expected, this sample had the highest level of initial spoilage, as indicated by its initial bacterial load of 8.16 log CFU/g by APC and 8.67 log CFU/g by LAB, both being the highest among all samples (Table 1).

### Temporal dynamics of fungal communities during spoilage in plant-based meat alternatives

**Fig 4:**
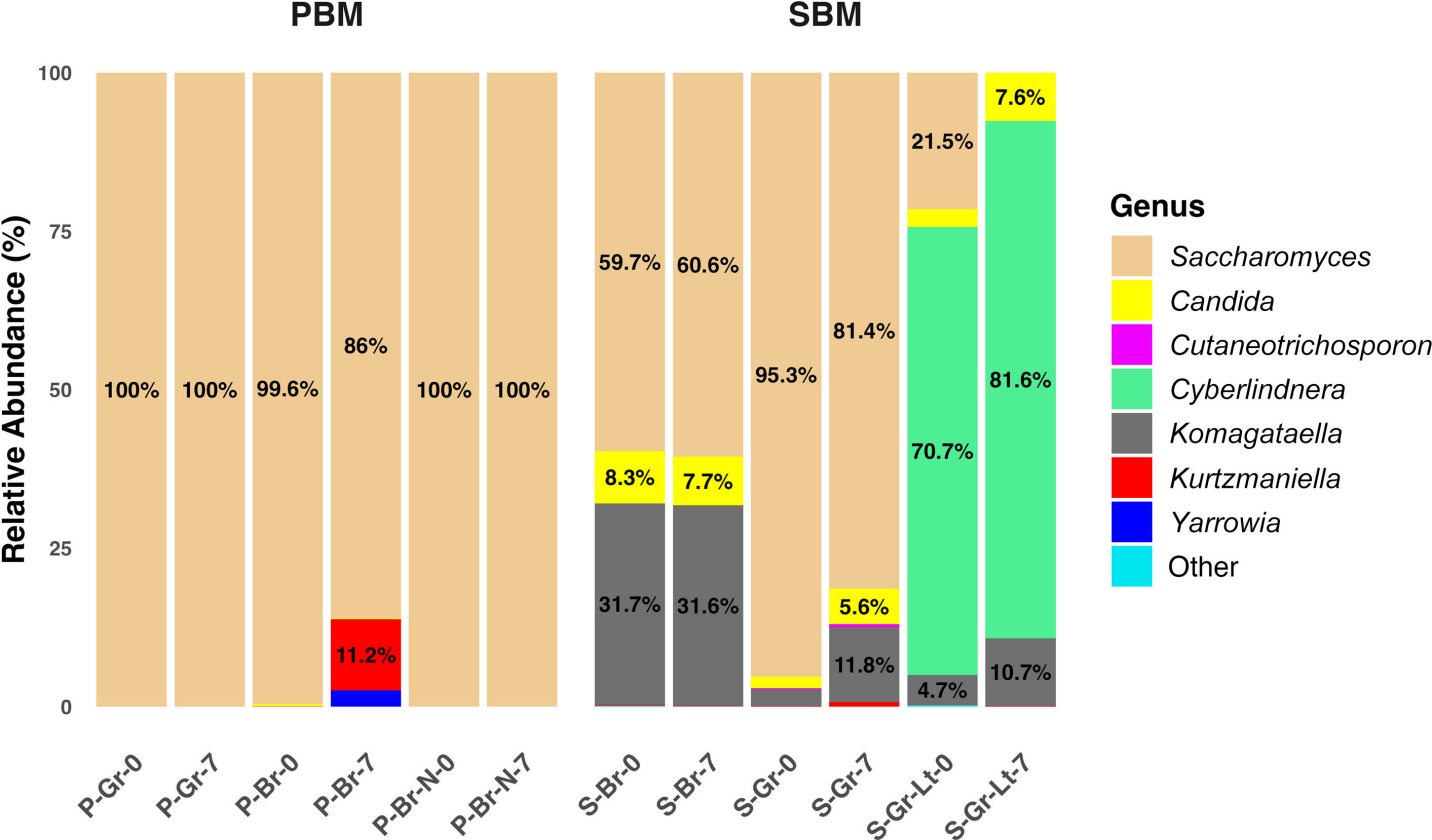
Indigenous microbial community composition of pea-based meat (PBM) and soy-based meat (SBM). Fungal taxonomic classification at the genus level. Percentages are only shown for relative abundances of 3% or more. P: Pea. S: Soy. Gr: Ground. Br: Burger. N: New size. Lt: Lite. 0: Sell-by date. 7: 7 days after the sell-by date.

Compared with bacterial communities, fungal communities in most PBMA samples showed considerably less compositional shift over 7 days of refrigerated storage (Fig. 3 and 4). A few exceptions include PBM-Burger (red samples, Fig. 3A) based on the Jaccard index and SBM-Ground-Lite (cyan samples, Fig. 3C) and SBM-Ground (blue samples, Fig. 3C) according to the Weighted UniFrac index.

Unlike other PBM samples, where *Saccharomyces* was the only detectable genus on both Day 0 and Day 7, PBM-Burger contained several low-abundance genera, comprising 0.4% of its fungal community on Day 0. Some of these genera increased considerably by Day 7, such as *Kurtzmaniella* and *Yarrowia* (Fig. 4).

In SBM-Ground, *Saccharomyces* (95.3%) was the dominant genus on Day 0, with low relative abundances of *Komagataella*, *Kurtzmaniella*, *Candida*, and *Cutaneotrichosporon*. By day 7, the relative abundance of *Saccharomyces* decreased to 81.4%, while the other genera increased. SBM-Ground-Lite exhibited a distinct fungal profile, with *Cyberlindnera* (70.7%) as the predominant genus on Day 0, alongside *Saccharomyces* (21.5%), *Komagataella* (4.7%), *Candida* (<3%), and rare taxa classified as “Other.” After 7 days, however, *Saccharomyces* and rare taxa became undetected as the relative abundances of other genera increased. In contrast, SBM-Burger was primarily composed of *Saccharomyces* (59.7%), *Komagataella* (31.7%), and *Candida* (8.3%), with minor contributions from *Kurtzmaniella* and rare taxa. These compositions remained relatively stable over time (Fig. 4).

### Spoilage microbiota succession appears to correlate with protein type.

Based on the Jaccard index, both bacterial and fungal communities in PBMAs exhibited distinct clustering in PCoA analysis by protein type (Fig. 1A and 3A). Regardless of sampling dates, the PBM and SBM clusters were separated along the PC1 axis, which explained the largest variation in the data. While this clustering was less pronounced using the Bray-Curtis and Weighted UniFrac indices (Fig. 1B, C, 3B and C), Adonis permutational multivariate analysis of variance (PERMANOVA) using Jaccard, Bray-Curtis, and Weighted UniFrac distance matrices indicated significant differences in bacterial composition (*P* = 0.001, *P* = 0.008, *P* = 0.015, respectively) and fungal composition (*P* = 0.001, *P* = 0.005, and *P* = 0.021, respectively) between the two protein groups (see Fig. S1 and S2 at https://doi.org/10.5281/zenodo.16950232).

The clustering of bacterial communities in PBMAs by protein type appeared to be further driven by the progression of spoilage. On Day 7, PCoA analyses based on both Jaccard and Bray-Curtis indices showed a divide between the PBM and SBM clusters along the primary axis (see Fig. S1A and B). This divide was attributable to the spoilage-mediated convergence of bacterial communities, which was observed across all samples but more pronounced in SBM samples. Notably, after 7 days of spoilage, the spread among SBM samples along both PCoA axes decreased substantially across all three indices (see Fig. S1A through C), whereas a similar reduction was observed for PBM samples only with the Jaccard index (see Fig. S1A). Consistent with this observation, regardless of the bacterial compositions of SBM samples on Day 0, they all became dominated by *Latilactobacillus* on Day 7 (Fig. 2).

**Fig 5:**
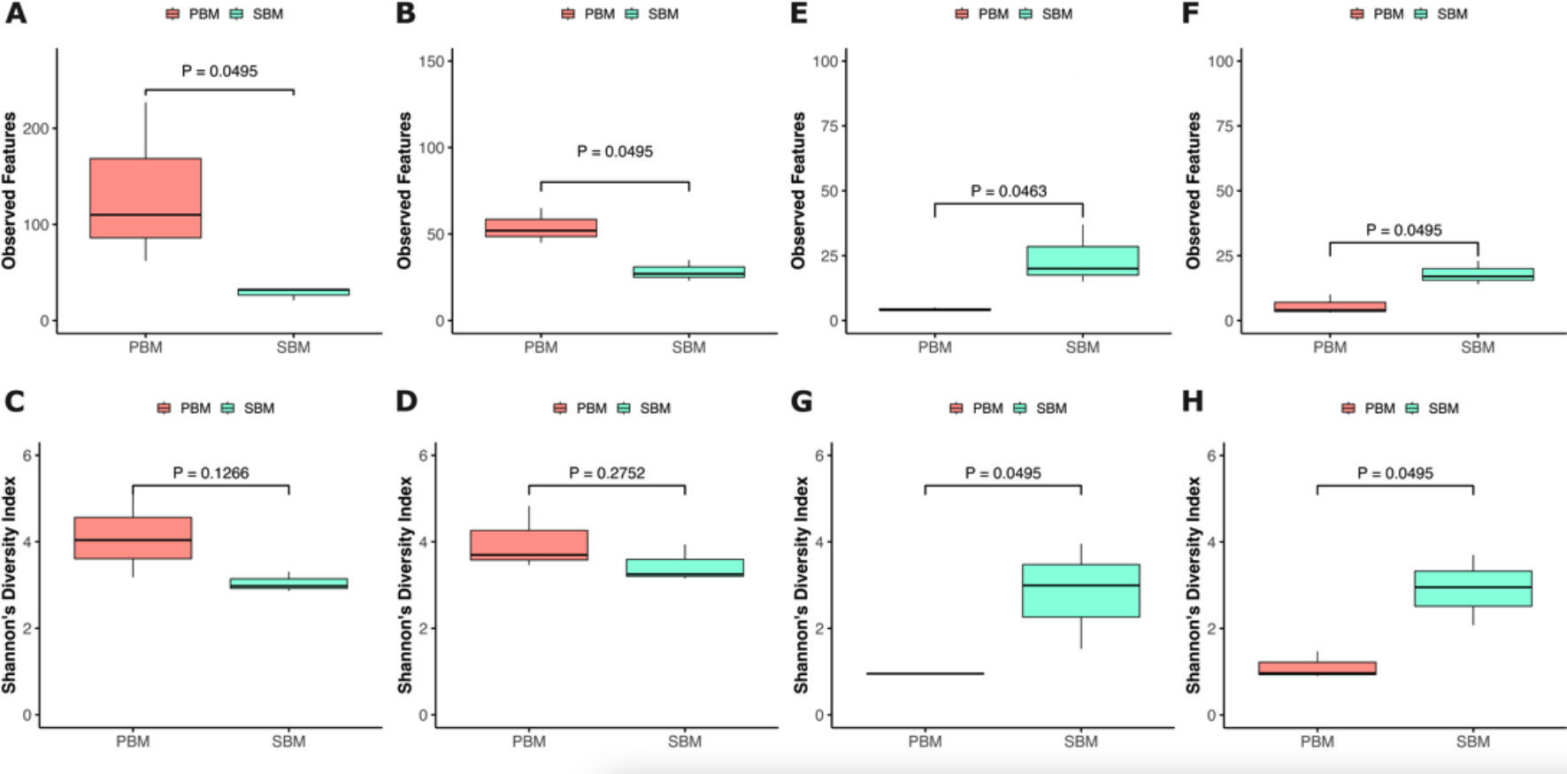
Alpha diversity of bacterial and fungal communities in plant-based meat alternatives (PBMAs). Observed features for bacterial communities are shown for Day 0 (**A**) and Day 7 (**B**), and for fungal communities on Day 0 (**E**) and Day 7 (**F**). Shannon diversity indexes for bacterial communities are shown for Day 0 (**C**) and Day 7 (**D**), and for fungal communities on Day 0 (**G**) and Day 7 (**H**). PBMs: Pea-based meats. SBMs: Soy-based meats.

Alpha diversity indices, including observed features and the Shannon index, were used to assess bacterial and fungal community richness and evenness in PBMs and SBMs. The Kruskal-Wallis rank-sum test revealed significant differences in bacterial communities between PBMs and SBMs based on observed features, which only measure community richness (*P* = 0.0495 for Days 0 and 7) (Fig. 5A and B). However, no significant difference was found in Shannon’s diversity, which measures both community richness and evenness (Fig. 5C and D). In fungal communities, the Kruskal-Wallis rank-sum test showed significant differences between PBMs and SBMs using both observed features (Day 0: *P* = 0.0463; Day 7: *P* = 0.0495) (Fig. 5E and F) and Shannon’s diversity (Day 0: *P* = 0.0495; Day 7: *P* = 0.0495) (Fig. 5G and H). PBMs exhibited higher bacterial richness than SBMs (Fig. 5). In contrast, fungal communities showed the opposite trend, with SBMs harboring greater fungal richness and diversity than PBMs (Fig. 4 and 5).

In addition to microbiota characteristics associated with protein type, the initial (Day 0) pH levels of the samples and their acidification during spoilage also varied by protein type. On the sell-by date (Day 0), the pH of PBMs ranged from 6.55 to 7.34, while that of SBMs ranged from 6.12 to 6.18 of SBMs (Table 1). The lower initial pH levels of SBMs were unlikely to be due to higher initial spoilage, as both mean LAB and APC loads in SBMs were lower than those of PBMs (Table 1). Over the course of a 7-day spoilage beyond the sell-by dates, the mean pH drop was greater in PBMs than in SBMs (Table 1). The initial relative abundances of lactic acid-producing bacteria, including *Leuconostoc*, *Latilactobacillus,* and *Brochothrix,* were higher in PBMs than SBMs, except for *Latilactobacillus-*rich SBM-Ground-Lite, which was likely more spoiled than other SBMs (Fig. 2). In comparison, the propionic acid-producer *Acidipropionibacterium* was unique to and relatively abundant in SBMs on Day 0 (Fig. 2). Interestingly, despite drastic compositional changes (Fig. 2) and substantial growth (Table 1) of its bacterial microbiota, SBM-Burger showed the smallest pH drop over 7 days (0.04). PBMs and SBMs exhibited similar water activities on Day 0 and Day 7, showing little change over the 7 days (Table 1).

## DISCUSSION

Our study characterized the temporal dynamics of the spoilage microbiota across various PBMAs (ground vs burger forms) during in-store display and subsequent simulated home storage. Marked differences in bacterial population levels were observed on their designated sell-by dates. These differences likely stemmed from variations in processing environments and post-manufacturing handling, which influence the initial microbial load. Factors, such as good manufacturing practices, raw material quality, hygiene protocols, and storage conditions, play a key role in shaping microbial dynamics (18, 51). Liu et al. (27) reported that immediately after thawing, SBM products contained bacterial populations of approximately 3 log CFU/g, while PBM products harbored about 4 log CFU/g. By the sell-by dates, the SBM items in our study (Burger and Ground) averaged ~4 log CFU/g, while PBM products (Ground and Burger-N) rose to ~6 log CFU/g. Notably, PBM Burger and SBM Ground Lite reached even higher bacterial loads, around 7.75 log CFU/g, than other PBM and SBM products on the sell-by date. Although our limited sample size cannot fully capture the variability of PMBA spoilage microbiota across the supply chain, the observed post-thaw increases and the wide variations in bacterial populations indicate inconsistencies in the post-manufacturing handling, including retail handling and storage of PBMAs in addition to the differences in processing environments. For example, food products under refrigerated display in retail environments can experience significant and varying degrees of temperature abuse due to display case design (open- and closed-door), sensor positioning, and the arrangement of products on the shelf (52).

By the end of the 7-day refrigerated storage, most bacterial populations in PBMAs reached approximately 9 log CFU/g—a spoilage level on par with those reported in refrigerated raw meats, such as poultry (53), fermented sausages (54, 55), cold-smoked salmon (56), fresh Greek Anthotyros whey cheese (57), and various fresh fruit and vegetable items (58, 59), where the proliferation of lactic acid bacteria typically drives spoilage. These findings suggest that PBMAs should be handled similarly to other perishable foods—by both retailers and consumers—to minimize temperature abuse and extend shelf life.

We characterized the temporal succession of spoilage microbiota in PBMAs with multiple Beta indices, providing a comprehensive and nuanced view of community change patterns. While the bacterial load present on the designated sell-by date was a primary determinant of subsequent community dynamics, a correlation was observed between community shifts and the underlying protein type of the PBMA. Notably, after 7 days of refrigerated storage, SBM samples exhibited a markedly tighter convergence of bacterial communities than PBM samples across all three Beta diversity indices, culminating in *Latilactobacillus* dominating every SBM sample. A recent study on PBMA products from Austrian supermarkets suggests that protein source (i.e., soy and pea) is not a main factor influencing microbial community patterns (28). This discrepancy likely reflects differences in study design: the earlier work captured single snapshots of microbial communities, whereas we characterized their temporal succession. Supporting our findings, Li et al. (29) likewise reported a distinct clustering of SBM and PBM samples after 7 days of refrigerated storage.

Distinct spoilage-community trajectories in SBM versus PBM products are likely traced back to differences in product formulation. Spoilage-related acidity reflects both the innate pH of ingredients and the acid-producing activities of the spoilage microorganisms. On their sell-by dates, PBM samples averaged about 6 log CFU/g and remained near-neutral pH, whereas SBM samples were more acidic and carried fewer spoilage bacteria (4 log CFU/g). These observations imply that SBMs are intrinsically more acidic than PBMs—probably because of their soy-based proteins versus the pea-based proteins in PBMs—consistent with the findings of Liu et al. (27). The neutral pH of PBMs may facilitate the early growth of spoilage bacteria after in-store product thawing, whereas the slightly acidic pH of SBMs may prolong such early growth.

In plant-based products that are formulated at or near neutral pH**,** *Leuconostoc* species usually seize the early spoilage niche, outpacing other bacteria by leveraging their broad metabolic versatility and rapid growth (60, 61). As spoilage proceeds and bacteria-produced acids accumulate, the decreasing pH suppresses these *Leuconostoc* populations that are more acid-sensitive, making way for more acid-tolerant species such as *Lactobacillus* (*Latilactibacillus*) (62–66) to assume dominance (62, 67).

By the end of our storage trial (Day 7), *Latilactibacillus* prevailed in SBM products but not in PMBs. Two factors likely drove the divergence. First, the intrinsically lower pH of soy formulations favors acid-resistant bacteria, such as *Latilactibacillus* species. Second, soy proteins are rich in arginine (68), which may allow *Latilactibacillus* to exploit the arginine deiminase (ADI) pathway, generating ATP and ammonia to counteract acidity and promote growth (69, 70). The expression of the *arc* operon that governs the ADI pathway peaks near pH 6.0 (69)**,** aligning with conditions during late-stage spoilage in SBMs. Together, these metabolic adaptations may give *Latilactobacillus* a competitive edge in acidic niches, explaining its recurring dominance in prolonged fermentations and spoilage events (71, 72).

The fungal profiles of SBMs and PBMs diverged as well. PBMs were dominated by *Saccharomyces*, with minor contributions from *Yarrowia*, *Candida*, and *Kurtzmaniella*. SBMs exhibited a richer assemblage that, in addition to *Saccharomyces* and *Candida,* included *Cutaneotrichosporon*, *Cyberlindnera*, *Komagataella*, and *Kurtzmaniella*. These differences likely stem from different raw materials, processing conditions, and occasional environmental contamination during production.

It is unsurprising to observe these fungal genera in PBMAs, as *Candida*, *Debaryomyces*, *Kluyveromyces*, *Pachysolen*, *Phaffia*, *Pichia* (*Komagataella*), *Saccharomyces*, and *Yarrowia* are widely recognized for their applications in food biotechnology (73). Specifically, yeast biomass and extracts, particularly from *Saccharomyces cerevisiae*, are frequently used in plant-based meat products to enhance flavor by imparting a meaty and umami taste (74). *Cyberlindnera jadinii* (Torula yeast), previously known as *Saccharomyces jadinii*, *Hansenula jadinii*, *Candida utilis*, *Pichia jadinii*, and *Lindnera jadinii*, is commonly used as a food additive in various food matrices and processed products, such as sausages and plant-based foods, due to its natural smoky and umami flavor (74, 75). Additionally, *Komagataella phaffii* (formerly *Pichia pastoris*) is intentionally incorporated into SBM formulations to produce leghemoglobins (LegH), which are key components responsible for replicating the meaty flavor and aroma of animal-based meats (76). The presence of *Cutaneotrichosporon* and *Yarrowia* in plant-based meat products can also be attributed to their applications in the food, cosmetic, and pharmaceutical industries. Notably, their ability to produce single-cell oils, which serve as sustainable alternatives to vegetable oils, likely explains their inclusion in these formulations (77, 78).

### Conclusion

Our results provide new insights into the temporal dynamics of the spoilage microbiota of PBMAs during retail display and extended storage, contributing to a deeper understanding of microbial behaviors in PBMAs. Microbial loads rose rapidly and heterogeneously across products after in-store thawing and past sell-by dates, underscoring the need to handle PBMAs like any other highly perishable food, both in stores and at home. Soy and pea-based matrices supported distinct spoilage trajectories. Follow-up work with model PBMA systems inoculated with defined spoilage consortia could help disentangle ingredient effects from other processing and product variables. Identification of ingredient-specific spoilage patterns may enable tailored and precision spoilage management for PBMAs. Future research incorporating sensory studies and multiomics approaches, such as metatranscriptomics and metabolomics, is needed to fully elucidate the microbial spoilage dynamics of PBMAs beyond spoilage microbiota profiling.

## Data Availability

Supplemental material has been deposited to Zenodo at https://doi.org/10.5281/zenodo.16950232.
